# Editorial: Trigger the microbiome changes in foods via metagenomic technologies: from diagnostic to potential changes in product safety or quality risk profiles

**DOI:** 10.3389/fbioe.2025.1766291

**Published:** 2026-01-02

**Authors:** Gabriela N. Tenea, Pablo Jarrin-V., Lin Lin

**Affiliations:** 1 Biofood and Nutraceutics Research and Development Group, Faculty of Engineering in Agricultural and Environmental Sciences, Universidad Tecnica del Norte, Ibarra, Ecuador; 2 Dirección de Innovación, Instituto Nacional de Biodiversidad, Quito, Pichincha, Ecuador; 3 School of Food and Biological Engineering, Jiangsu University, Zhenjiang, China

**Keywords:** antibiotic resistant genes (ARG), food quality, food safety, fruits, long-read metagenomics, metabarcoding, metal resistant genes (MRG), next-generation sequencing

From raw agricultural inputs to the finished consumer product, the microbial communities of our food production systems are a crucial factor in determining both quality and safety. For many years, food safety diagnostics relied on labor-intensive, culture-based techniques that only captured a small portion of the microbial reality. However, the integration of metagenomic technologies to accurately characterize entire food microbiomes is on the verge of a disruptive revolution, as demonstrated by the studies gathered in this Research Topic. The use of metagenomic technologies offers unmatched chances for proactive risk assessment and quality control by taking us beyond straightforward hazard detection and toward a comprehensive understanding of the microbial mechanisms that determine the quality of our food.

Metabarcoding, shotgun metagenomics, and long-read sequencing platforms provide high-resolution, culture-independent frameworks for profiling microbial communities. These tools are transforming food safety research by enabling detailed analysis of community dynamics in complex biochemical environments such as cacao fermentation, as revealed by Tigrero-Vaca et al. Their multi-platform metagenomic strategy demonstrated the potential for future *in situ* monitoring while providing high-resolution taxonomic and functional profiles. Using hybrid assembly approaches, Tigrero-Vaca et al. mapped functional genes linked to both primary carbohydrate metabolism and secondary metabolite pathways, including those associated with polyphenol transformations that contribute to chocolate flavor precursors. With efficient and appropriate bioinformatic frameworks, the vast trove of information, offered by food-applied metagenomics, guarantees quicker response times during foodborne outbreaks and permits rapid detection and tracking of microbiological pathogens, improving public health outcomes and lowering the need for expensive recalls Tigrero-Vaca et al.

Yet, challenges also arise with different technologies. Tigrero-Vaca et al. directly compares Illumina sequencing with the *in situ* monitoring potential of Nanopore MinION. While both platforms confirmed the overall microbial succession, the authors uncovered critical, platform-specific biases at the species level. This finding powerfully illustrates the core challenge highlighted in Tigrero-Vaca et al. the urgent need for standardized bioinformatic workflows to resolve such discrepancies and take the best from each available technology.

Metagenomics is useful for much more than just diagnostics; it gives us the information we need to better understand the fundamental processes that underlie the emergence and spread of microorganisms. With microbial interactions in mind, metagenomics enables a system-wide understanding of interrelationships rather than approaching food safety as a linear “hazard-by-hazard” problem. This ecological viewpoint is exemplified by the study on fungal-dominated microbiomes by Zhao et al. They found significant differences between bacterial-rich and fungal-rich fermentation environments in terms of community composition and structure, functional potential, and, most significantly, the resistome. A crucial hypothesis is raised by the discovery that fungal dominance was associated with a lower abundance of Antibiotic Resistance Genes (ARGs) (Zhao et al.); implying that ecological factors can actively prevent the spread of ARGs. This insight is paramount, as it can guide the design of more focused and successful interventions that leverage a food’s native or introduced microbiome to mitigate the spread of AMR.

A key strength of the contributions forming this Research Topic is the focus on functional metagenomics, moving beyond simple taxonomic cataloging to understand what these microbes are doing. The work by Li et al. in rice-flavor Baijiu provides a clear model for this, correlating the succession of specific genera like *Lacticaseibacillus* with functional KEGG pathways responsible for the biosynthesis of volatile flavor compounds, the study provides criteria for improving starter cultures and thus product quality and consistency.

In summary, the four manuscripts that make this Research Topic, allow us to propose that metagenomic technologies are a keystone for an upcoming technological change in how we manage food quality and safety. By offering a system-wide, mechanistic understanding of the food microbiome, metagenomic studies go beyond diagnostics. In particular:

1. Risk Assessment: Metagenomics facilitates proactive risk assessment by revealing dynamic ecological factors, such as the relationship between fungal dominance and the suppression of ARGs (Zhao et al.), guiding targeted interventions to limit resistance spread.
2. Quality Control: Metagenomics establishes a direct microbe-to-flavor correlation (Li et al.), offering the scientific basis needed to design optimized, robust starter cultures for consistent and enhanced food quality.
3. Technological Validation: Studies confirm the relevance of a diverse array of genomic platforms and techniques, including the possibility of *in situ* monitoring (Tigrero-Vaca et al.). These diverse Research Topic of metagenomic applications and technologies emphasize the critical need for validated and standardized workflows to translate these powerful tools from a laboratory environment into industry-wide risk management protocols and regulatory frameworks (Tigrero-Vaca et al.).

Finally, the contributions of this Research Topic points to the future. While the need for standardized workflows is a recurring theme, the review by Tigrero-Vaca et al. highlights what comes next. As these technologies generate increasingly massive and complex datasets, the integration of Artificial Intelligence (AI) and Machine Learning (ML) will become essential for predictive modeling of pathogen risk or fermentation outcomes. Furthermore, these techniques are expanding into new domains, such as the use of metabarcoding for food authentication to combat fraud and ensure traceability. This Research Topic thus provides a snapshot of a field in rapid transition: from cataloging microbes to understanding their function, and from reactive diagnostics to a predictive, system-wide science.

